# Tier-based standards for FAIR sequence data and metadata sharing in microbiome research

**DOI:** 10.1093/nar/gkaf777

**Published:** 2025-08-18

**Authors:** Lina Kim, Anton Lavrinienko, Zuzana Sebechlebska, Sven Stoltenberg, Nicholas A Bokulich

**Affiliations:** Department of Health Sciences and Technology, ETH Zurich, 8092 Zurich, Switzerland; Department of Health Sciences and Technology, ETH Zurich, 8092 Zurich, Switzerland; Department of Health Sciences and Technology, ETH Zurich, 8092 Zurich, Switzerland; Department of Health Sciences and Technology, ETH Zurich, 8092 Zurich, Switzerland; Department of Health Sciences and Technology, ETH Zurich, 8092 Zurich, Switzerland

## Abstract

Microbiome research is a growing, data-driven field within the life sciences. While policies exist for sharing microbiome sequence data and using standardized metadata schemes, compliance among researchers varies. To promote open research data best practices in microbiome research and adjacent communities, we (i) propose two tiered badge systems to evaluate data/metadata sharing compliance, and (ii) developed an automated evaluation tool to determine adherence to data reporting standards in publications with amplicon and metagenome sequence data. In a systematic evaluation of publications (*n* = 2929) spanning human gut microbiome research, and case studies of soil and gut microbiota (*n* = 370), we found nearly half do not meet minimum standards for sequence data availability. Moreover, poor standardization of metadata creates a high barrier to harmonization and cross-study comparison. Using this badge system and evaluation tool, our proof-of-concept work exposes (i) the ineffectiveness of sequence data availability statements and (ii) the lack of consistent metadata reporting. We highlight the need for improved practices and infrastructure that reduce barriers to data submission and maximize reproducibility in microbiome research. We anticipate that our tiered badge framework will promote dialogue regarding data sharing practices and facilitate data reuse, supporting best practices that make microbiome data Findable, Accessible, Interoperable, and Reusable (FAIR).

## Graphical Abstract

**Graphical Abstract:**
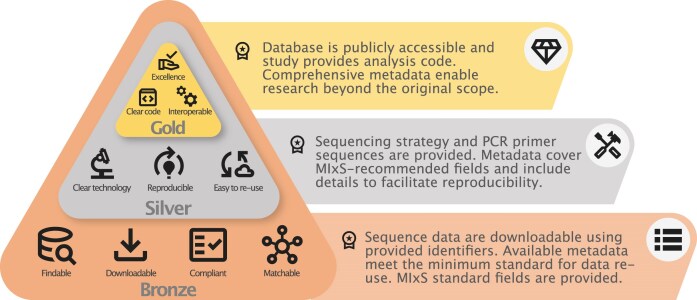

## Introduction

Reproducibility and data reuse are fundamental to advancing scientific discovery and innovation, yet both remain persistent challenges across the life sciences [1]. Open research data (ORD) are publicly accessible, usable, and redistributable datasets. Ideally, ORD should also adhere to Findable, Accessible, Interoperable, and Reusable (FAIR) [2] principles and best practices that facilitate data reuse in research. Such openness is crucial in the life sciences, which are increasingly reliant on large-scale, high-dimensional datasets. Nearly half of all researchers use data generated by other scientists [3], and this number is likely to grow due to the increasing adoption of computational methods in all areas of research [4, 5]. However, data reuse across research disciplines is hindered by subpar sharing practices including poor accessibility and interoperability, and particularly insufficient data and metadata reporting [6].

High-throughput DNA sequencing technologies have revolutionized research across all domains of the life sciences and over the past two decades have generated tremendous amounts of data [7]. A prominent application of these technologies is the study of microbiomes: microbial communities, their components, products, interactions, and functional activities [8, 9] that play important roles in both host-associated and environmental ecosystems [10–13]. Access to the raw sequence data and metadata is key to scientific transparency and innovative reuse, yet data accessibility [14] and metadata standardization issues [15] are salient in microbiome research.

Several data sharing standards already exist for depositing the raw microbiome sequence data, including the INSDC repositories [16, 17] as well as several microbiome-specific databases with integrated analytical workflows (e.g. Qiita [18], MGnify [19], and NMDC [20]). Similarly, various metadata standards were developed to establish a controlled vocabulary for sample collection, preparation, and data processing methods [21, 22]. The Genomic Standards Consortium (GSC) established the Minimum Information for any (x) Sequence standards (MIxS), with specific checklists for marker-gene [15] and metagenome data [23]; these are currently supported by major sequence data repositories, including INSDC. Despite such institutional efforts, compliance with these data sharing standards among researchers is still limited [22].

Consequently, an increasing number of microbiome studies fail to meet standards and hamper the scientific community’s efforts toward open data sharing [24]. Numerous studies either provide no accessible sequence data or deposit the raw sequence data with insufficient or mismatched metadata that can not effectively be linked to the provided sequence data [25–27]. This is a wider problem across numerous scientific disciplines, with publication data often remaining incomplete [28, 29]. According to estimates, only a quarter of papers published in *Science* meet computational reproducibility [30] despite its policy requiring sharing of “all data necessary to understand, assess, and extend the conclusions of the manuscript.” Particularly frustrating is the refusal of authors to share published data despite reporting those as “available upon request” [31, 32]. Indeed, the vast majority of researchers are not compliant with their own published data availability statements (DAS): of nearly 1800 reviewed publications with DAS suggesting data availability upon “reasonable request,” a mere 7% of authors provided usable data [33].

Although depositing raw data prior to publication is now mandated by most journals and funding agencies, the key issue is that mechanisms for assessing and validating data reporting practices are largely missing [34]. We propose a two-pronged solution to address these issues and promote reproducibility in the field of microbiome science: (i) tier-based standards to assess reproducibility of publications by evaluating data accessibility statements and metadata, and (ii) tools to programmatically determine adherence to these tiered standards, enabling evaluation prepublication (e.g. during review) or postpublication (e.g. for selection during meta-analysis).

## Materials and methods

### Tier-based standards for sequence data and metadata sharing

We propose two tiered badge systems to evaluate the openness of research data within the microbiome research community: one for sequence data reporting, and one for metadata reporting. These parallel standards are linked to provide effective data availability reporting for both nucleotide sequence data and associated metadata (Graphical abstract and Tables 1 and 2). We consider in this framework the availability of next-generation sequencing (NGS)-based data, representing sequences comprising microbial communities (in the microbiome field, these are microbial marker-gene amplicon and metagenome sequences). However, we anticipate that this framework can be extended and adopted by other fields within the life sciences relying on marker-gene (amplicon) data, such as for instance environmental DNA and diet metabarcoding research.

**Table 1.** Requirements for nucleotide sequence data reporting

Requirement Type	Bronze	Silver	Gold
Data accessibility	Sequence data are downloadable and not paywalled	Sequence data are downloadable and not paywalled	Sequence data are downloadable and not paywalled
Data accessibility	Accession numbers provided	Accession numbers provided	Accession numbers provided
Data accessibility	Raw sequences given as BCL or FASTQ	Raw sequences given as BCL or FASTQ	Raw sequences given as BCL or FASTQ
Sample processing metadata		Sequencing method provided: marker gene or metagenomics	Sequencing method provided: marker gene or metagenomics
Sample processing metadata		PCR primer sequences	PCR primer sequences
Data accessibility			If data are not public, DAS clarifies access requirements
Data accessibility			Database is publicly accessible, with no login and/or institutional affiliation, e.g. SRA or Figshare
Data processing metadata			Code provided

Bronze represents a minimal set of requirements for releasing open data, while the Gold tier represents both open and FAIR research data for easy reuse.

**Table 2.** Example of requirements for metadata reporting

MIxS v.6.0 Source	Bronze	Silver	Gold	MIxS requirement
MIxS core	Sample name	Sample name	Sample name	Mandatory^a^
MIxS core	Project name	Project name	Project name	Mandatory
MIxS core	Taxonomy ID of DNA sample	Taxonomy ID of DNA sample	Taxonomy ID of DNA sample	Mandatory^a^
MIxS core	Collection date	Collection date	Collection date	Mandatory^a^,^a^
MIxS core	Geographic location (latitude/longitude)	Geographic location (latitude/longitude)	Geographic location (latitude/longitude)	Mandatory^a^,^a^
MIxS core	Geographic location (country/region)	Geographic location (country/region)	Geographic location (country/region)	Mandatory^a^
MIxS core	Broad-scale environmental context	Broad-scale environmental context	Broad-scale environmental context	Mandatory
MIxS core	Local-scale environmental context	Local-scale environmental context	Local-scale environmental context	Mandatory
MIxS core	Environmental medium	Environmental medium	Environmental medium	Mandatory
MIxS core		Sequencing method	Sequencing method	Mandatory
MIxS core		Negative control type	Negative control type	Optional
MIxS core/MIMARKS checklist		Target gene	Target gene	Mandatory
MIxS core/MIMARKS checklist		PCR primers	PCR primers	Optional
MIxS human-gut package		Host subject ID	Host subject ID	Optional
MIxS human-gut package		Host disease status	Host disease status	Optional
MIxS human-gut package		Host total mass	Host total mass	Optional
MIxS human-gut package		Host sex	Host sex	Optional
MIxS core			Sample material processing	Optional
MIxS core			Nucleic acid extraction	Optional
MIxS human-gut package			Host height	Optional
MIxS human-gut package			Host diet	Optional
MIxS human-gut package			Host occupation	Optional

Bronze represents a minimal set of requirements for releasing open data, while the Gold tier represents both open and FAIR research data for easy reuse.

Note that while in the example below the tiers do include the fields of a study that can be used to describe an experiment, or set of experiments, in an increasingly detailed manner (from Bronze to Gold), thus enabling data reuse, reproducibility, and eventually, research beyond the original scope; Silver and Gold are somewhat study-specific, i.e. these tiers do not assume the ever-increasing number of reported MIxS fields, but rather their quality and value for reproducibility and future data reuse. The MIxS-specific fields are based on the MIxS v6.0 release (https://www.ebi.ac.uk/ena/browser/view/ERC000015).

^a^Mandatory field in the NCBI metagenome or environmental package (https://submit.ncbi.nlm.nih.gov/biosample/template/).

^b^Mandatory in the ENA default sample checklist (https://www.ebi.ac.uk/ena/browser/view/ERC000011).

An ORD-compliant dataset requires that (i) the unprocessed sequence data and standardized metadata are available, ideally in standard formats [15], and (ii) both are downloadable from a public repository or a database. The US National Center for Biotechnology Information (NCBI)’s Sequence Read Archive (SRA) and European Bioinformatics Institute (EBI)’s European Nucleotide Archive (ENA) are excellent examples of databases preserved in the public domain, providing “free, unrestricted, and permanent access to the data” [35].

For a dataset to be FAIR, additional properties are necessary beyond sequence data and metadata deposition. For example, to reproduce the publication’s analysis, knowledge of the conducted sequencing method (e.g. marker-gene versus metagenomic sequencing) is necessary as bioinformatics analyses vary substantially and can be quite distinct [36] and/or require additional context. Notably, if the dataset was produced with marker-gene sequencing, primer sequences are necessary as primer choice affects sequence capture, database choice, and taxonomy identification [37, 38]. Finally, full reproducibility and reuse of the raw dataset is only possible with the availability of the high-quality metadata and executable code used for the original analysis [39].

The badge system for sequence data and metadata availability standards ranks publications by integrating well accepted—but not yet universally adopted—standards:

1. Bronze: Available data meet the minimum standard for data reuse. To meet this standard, the publication includes basic details such as the accession ID for the associated sequence data and the metadata that provides environmental context for given samples. With these, researchers are able to find and, when suitable for their purpose, use data from the original publication.
2. Silver: Facilitated reproducibility; this is a balance between comprehensiveness and feasibility for most researchers. This standard integrates more comprehensive metadata with additional fields such as recommended fields in the MIxS checklists, and other domain-specific standards. Sequence data in this tier are not necessarily openly accessible, but the publication clarifies access requirements for reuse.
3. Gold: These data provide valuable information to address research questions beyond the original scope. With freely and publicly accessible sequence data, any researcher is able to use these data without barriers. And with additional optional metadata details from specific MIxS environmental packages, researchers are able to effectively characterize this dataset for deeper investigation.

### Automated badge evaluation tool

To automatically assess ORD reporting standards, we developed software to evaluate the openness of a given research publication. This software package is intended as a proof of concept for assessing the feasibility of automated screening of the nucleotide sequence data accessibility badge system described above (Table 1). The Python package MISHMASH (MIcrobiome Sequence and Metadata Availability Standards) automatically evaluates the openness of nucleotide sequence data reporting. It assesses given articles for the required reporting components listed in Table 1, and uses this information to assign a badge (Bronze, Silver, or Gold). MISHMASH was developed as an open-source Python 3 package under the BSD-3-Clause license, and is available at https://github.com/bokulich-lab/mishmash (also deposited in Zenodo: https://zenodo.org/records/16039307).

With two main commands, users can evaluate data reporting in PubMed Central (PMC) publications. The command assess_sequences takes as input a space-separated list of PMC IDs or a file containing PMC IDs, where each ID corresponds to a single PMC publication. For each publication, the package returns a sequence accessibility badge (Bronze, Silver, Gold, or “Cannot be determined”) based on the criteria in Table 1 and outputs the following components as a comma-separated values file (and indicates whether these components were not detected as transparent criteria for the badge assessment):

- INSDC database associated with the uploaded sequence data i.e. SRA, ENA, or DDBJ.
- INSDC accession numbers corresponding to the sequence data uploaded to INSDC databases.
- Number of sequence records (INSDC runs) associated with the input article.
- Primer sequences of amplified variable regions, if an amplicon-based study.
- Code availability per study.

The command assess_metadata retrieves metadata associated with a sequence record from an INSDC database. It returns the entirety of the metadata record as a comma-separated values file. If a particular GSC checklist or environmental package is used (e.g. MIMARKS or MIMS metagenome/environmental and human gut), its name is returned as reported to the INSDC.

### Case studies to test and validate evaluation tool

To assess the effectiveness of our standards and software, we manually validated the MISHMASH program and sequence data availability tiers on three case studies:

- Case Study 1: a cross-section of human gut microbiome publications released between 1 January 2024 and 30 June 2024 (*n* = 85).
- Case Study 2: a list of human gut mycobiota publications from 2013 through 2023 (*n* = 199).
- Case Study 3: a cross-section of soil microbiome publications released between 1 January 2023 and 31 December 2023 (*n* = 86).

Publications were screened on the NCBI PubMed database for peer-reviewed, original research papers with licenses allowing for full-text automated retrieval. The full PubMed search queries can be found with their corresponding PRISMA diagrams (generated with [40]) in the Supplementary Figs S1–S3. Each publication was manually evaluated for a badge assignment using the proposed tier criteria presented in Table 1.

For effective future evaluations of large datasets, we established a baseline accuracy rate for the automated badge prediction algorithm MISHMASH. By comparing manual badge assignments with programmatically generated predictions for each of the 284 publications, we established a baseline accuracy rate for the automated badge prediction algorithm. Accuracy is reported as overall accuracy (sum of correct predictions over all predictions), macro-average F1, and overall weighted F1 score [41].

To investigate current metadata sharing practices, we manually examined 42 publications with INSDC-based datasets from Case Study 1. We extracted metadata associated with each article’s INSDC entry to evaluate badges for metadata reporting.

### Open field survey of human gut microbiome literature

**Figure 1.**
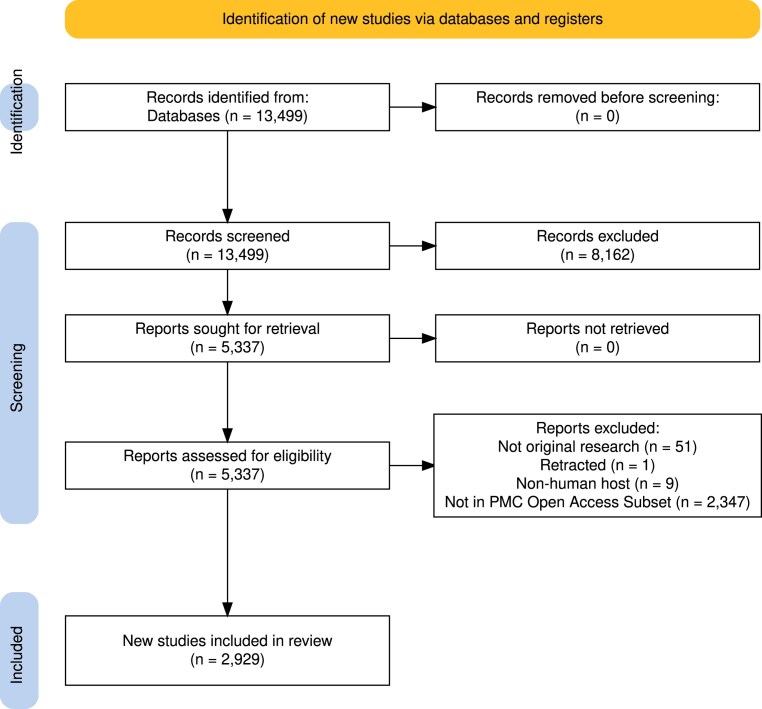
Overview of the workflow used for selection of publications for the open field survey. Records were removed during the initial screen (*n*= 8162) for non-English text or nonhuman subjects based on MEDLINE indexing.

To evaluate the openness and FAIRness of the microbiome research field, we compiled an open field survey dataset of human gut microbiome publications comprising 2929 articles published between 2003 and 2023 (Fig. 1). As in the above case studies, included publications were peer-reviewed, original research papers with licenses allowing for automated full-text retrieval. With the MISHMASH algorithm, we estimated the comprehensive state of data sharing from the past two decades of human gut microbiome literature.

## Results

### Manual error rate calculation demonstrates high accuracy

Using the studies in Case Studies 1 and 2, we manually evaluated each publication’s badge assignment based on the tiered requirements established in Table 1 (Supplementary Tables S1 and S2). In both manually evaluated case studies, the most populated category was None, of publications that do not meet the minimum standards for open sequence data. The vast majority of remaining publications for both case studies were assigned the Silver badge, with similar proportions of both Bronze and Silver assignments.

As Case Study 2 spans 10 years of publications, we additionally explored temporal patterns in data availability. The primary trend is of total frequency (Fig. 3B). The number of annual publications steadily increases from 1 article in 2013 to 53 by the end of 2023. This is possibly explained by the decreasing cost of sequencing and growing interest in gut fungi in relation to human health. The proportion of publications without a badge assignment (Nones) also slowly decreases over time (Fig. 3A). We also investigated the potential influence of the article publisher on data openness. Of the 199 publications analyzed in Case Study 2, 153 (76.9%) were published in journals whose publishing groups included 10 or more publications from our list. Among these, data accessibility varies widely by publisher (Supplementary Fig. S5 and Supplementary Table S3). The proportion of published articles with no publicly available sequence data ranges from 18% to 44%.

Tested on the combined dataset of the aforementioned case studies, the automated badge predictor correctly classified a publication’s assigned badge at an overall accuracy of 84.0% (Table 3). The discrepancy between the macro-average and weighted F1 scores (0.79 versus 0.84) indicates varying error rates across different categories. The badge category Gold has the lowest support proportion (9.9%) with an F1 score of 0.62, while the Silver category with the highest support proportion (38.7%) has an F1 score of 0.82.

We also examined the accuracy rates for a more generalized classification of None versus Badged (Bronze, Silver, and Gold) publications. This is relevant to cases in which researchers search for publications with minimally available sequence data. For this binary badge predictor, our automated algorithm evaluates with an overall accuracy of 97.2%.

### Metadata survey demonstrates poor harmonization

Of the 42 Case Study 1 publications with data uploaded to the INSDC, only 21 of these datasets used an MIxS metadata scheme. Those remaining submitted INSDC-required minimal metadata (e.g. SRA/ENA-specific checklists); all datasets also included additional attributes relevant to each study.

**Figure 2.**
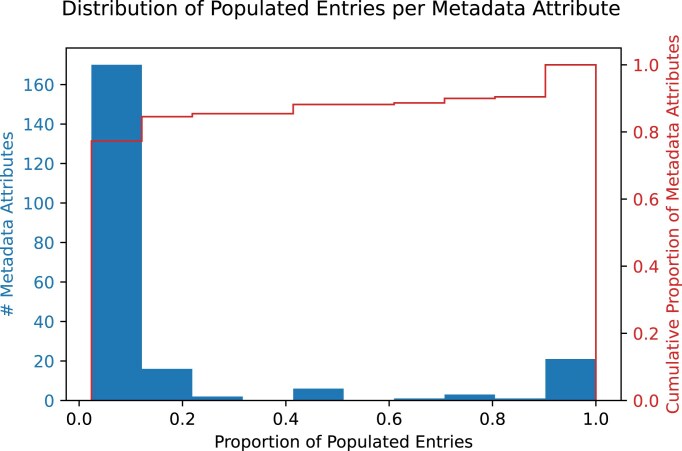
A histogram of metadata comprehensiveness across 42 studies and 220 metadata attributes. The proportion of populated entries indicates the percentage of publications with submitted metadata for a given attribute. For instance, 167/220 metadata attributes are populated at a rate of 10% or less, i.e. up to 90% of studies do not use or leave those metadata attributes blank.

While some metadata attributes such as Organism are complete for every INSDC-submitted sample, others are less commonly utilized (Supplementary Table S4). Sample names are submitted to the INSDC through seven different terms depending on the study and data submitter, such as “Sample Name” and “User_sample_ID.” Sample environmental contexts and microbiome host identifiers are likewise spread across variously named metadata attributes. In all, the union of metadata attributes from 42 studies totaled 220. Only 16 of these attributes were consistently filled across every study, and nearly 80% of metadata attributes had entries from fewer than eight publications (Fig. 2). The median attribute included metadata from only a single study (2.4%), showcasing the immense sparsity of poorly labeled metadata.

### Open field survey and case studies show poor sequencing data accessibility

**Figure 3.**
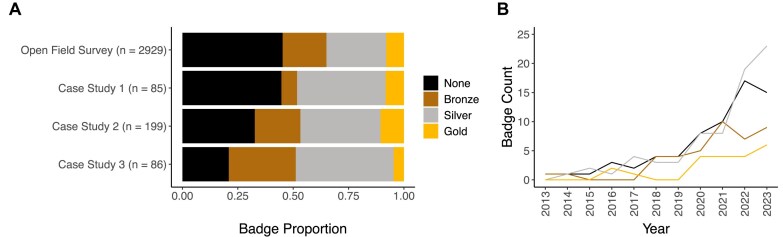
Trends of low open data compliance in microbiome literature. (**A**) Predicted badge assignments for an open field survey of human gut-associated microbiome publications published from 2003 through 2023 (*n*= 2929), and two smaller case studies with ground truth validation: badge assignments for human gut-associated microbiome publications published from January through June 2024 (*n =*85), badge assignments for human fungal gut microbiome (mycobiome) publications published from 2013 through 2023 (*n =*199), and badge assignments for soil microbiome publications published from January through December 2023 (*n =* 86). (**B**) A temporal look at badge assignments for Case Study 2, by total frequency per year.

An open field survey of DAS found in microbiome research revealed three consistent trends: (i) nearly half of all publications (45.2%) did not meet the minimum standards for open sequence data (Nones), (ii) on the other extreme, only a few publications (8.0%) qualified for the Gold badge, and (iii) publication with the Silver badge (27.0%) outnumbered those with Bronze and Gold—consistent with our expectation that the Silver standard represents a balance between comprehensiveness and feasibility for many researchers. Our survey (Fig. 3) yielded remarkably similar estimates to manual badge assignments for Case Studies 1 and 2, further reinforcing these findings and suggesting that such data reporting practices (i.e. high rates of missing sequence data) are consistent at the repository scale.

Interestingly, these trends likely reflect distinct user communities: researchers who simply share no data or report that data are “available upon request,” and researchers who exceed the minimum requirements, aiming for a higher standard of openness that balances availability with feasibility, as indicated by higher inclusion rates for Silver versus Bronze badges in all case studies and our large-scale literature survey (Fig. 3).

An evaluation of a completely separate research area—soil microbiota, as Case Study 3—yielded similar proportions of data accessibility according to our badge system (Fig. 3). In a pattern similar to those of human microbiome case studies, the proportion of Silver badges had the greatest plurality at 38/86 (44.2%) and Gold the smallest fraction at 4/86 (4.7%) of all publications. Case Studies 1 and 2 had Silver badge fractions of 40.0% and 35.5%, respectively, and both had Gold-assigned papers as the smallest fractions at 8.2% and 10.4%. Case Study 3 had a far smaller proportion of None-badged publications than any of the previous case studies, at 20.9% compared with 44.7% (Case Study 1) and 33.3% (Case Study 2).

The error rates reported for our automated algorithm MISHMASH (Table 3) remain similar for this previously untested dataset. The overall accuracy of this four-category badge algorithm is 91.9%, greater than the 84.0% from Case Studies 1 and 2 combined (*n =*268). The macro-average F1 and weighted F1 scores are both greater for Case Study 3, at 81.1% and 92.5%, respectively. The overall accuracy for binary classification is comparable for Case Study 3, at 96.5% versus 97.4% for Case Studies 1 and 2, respectively.

**Table 3.** Accuracy rates for the automated badge prediction algorithm in establishing sequence data availability

	Four-category badge prediction (None, Bronze, Silver, and Gold)	Binary badge prediction (None versus Badged)
Overall accuracy	0.84	0.97
Macro-average F1	0.79	0.97
Weighted F1	0.84	0.97

### INSDC databases compose the bulk of reported sequence data

Microbiome publications upload their sequence data to numerous repositories. Among publications with associated sequence data (i.e. a badge of Bronze, Silver, or Gold), the majority (∼65%) utilized the NCBI SRA to store and share their data (Table 4). The ENA, another INSDC database, is the second-most frequently used, representing ∼18% of total publications. The third INSDC database, the DNA Data Bank of Japan, falls at similar proportions to the China National Center for Bioinformation’s National Genomics Data Center (CNCB-NGDC) [42] used by ∼3% of publications screened.

**Table 4.** Distribution of utilized databases among microbiome publications

Database	Publications in Case Studies 1–3 (%)	Publications in open field survey (%)
NCBI SRA	151 (64.53%)	1053 (65.69%)
ENA	42 (17.95%)	289 (18.03%)
CNCB-NGDC	8 (3.42%)	52 (3.24%)
DNA Data Bank of Japan	8 (3.42%)	35 (2.18%)
MG-RAST	6 (2.56%)	40 (2.50%)
Figshare	5 (2.14%)	28 (1.75%)
Other	4 (1.71%)	31 (1.93%)

The “Other” category includes Zenodo, the European Genome-Phenome Archive (EGA), and the Chinese National GeneBank Sequence Archive. Publications uploading data to multiple databases comprise fewer than 5% of publications and were not included.

Much lower proportions of studies used the metagenome-specific platform MG-RAST (Metagenomic Rapid Annotations using Subsystems Technology) and Figshare (a surprising observation, as this repository is not specific to sequence data). Less frequently used are platforms such as Zenodo, the European Genome-Phenome Archive (EGA), and the Chinese National GeneBank Sequence Archive; these platforms each composed <1% of all publications each.

**Figure 4.**
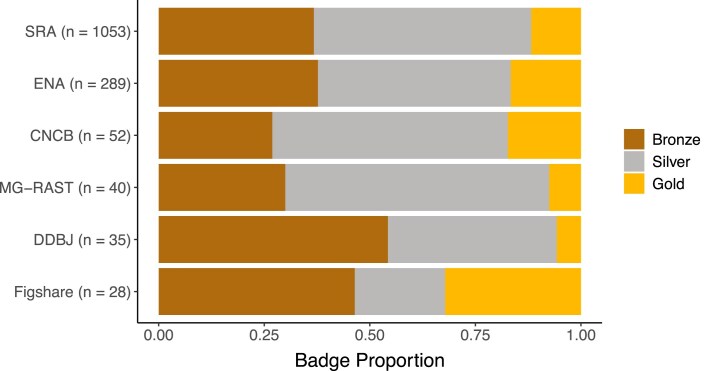
Distribution of badge assignments among utilized databases among papers from the open field survey.

On the other hand, the distribution of badge assignments among utilized sequence repositories remained quite consistent. Publications from the open field survey (*n =*2929) aggregated similarly, with Bronze/Silver/Gold proportions among the most frequently used archives (SRA and ENA) standing nearly identical (Fig. 4).

## Discussion

### Systematic issues in current metadata sharing practices

Despite the critical role of metadata in microbiome research, standardized metadata are not commonly available, leaving microbiome datasets incomplete and limiting their utility [34]. Indeed, most articles in our Case Study 1 did not qualify for a badge assignment when considering the reported metadata. This contrasts with the DAS for the same publications, where about half of the screened papers met eligibility for Bronze, Silver, or even Gold badges (Fig. 3A). Metadata records are generally more complex and can be difficult to evaluate automatically using scraper tools. However, our implementation relies on a set of requirements for metadata reporting defined by the Genomics Standards Consortium’s MIxS checklists that should be standardized and machine readable in a compliant report [15]. Although adoption of the MIxS metadata standards is widespread across the microbiome community [22], metadata associated with most samples in INSDC databases remain poorly standardized, limiting the potential for microbiome data reuse. We found that the practice of adding nonstandard metadata leads to considerable variation, making it challenging to harmonize metadata across studies.

More broadly, our findings highlight several systematic issues in current metadata collection and sharing practices. For example, several specific problems hinder efforts to evaluate existing metadata using the badge system alongside the DAS: (i) metadata often lack standardization and are reported without controlled vocabulary or standardized terminology, (ii) similar information is recorded in different fields across studies, and (iii) information is reported, but the MIxS package requirements are applied incorrectly. In our case studies, the diversity of terms used to describe similar variables increased the dimensions of reported metadata, making it challenging to assess reporting practices automatically (Fig. 2). More importantly, these same issues create barriers to data reuse, as it can be difficult or even impossible to harmonize such metadata records across studies without extensive manual inspection and curation. Similarly, a recent survey of public microbiome data identified over 3000 different descriptors used to report common elements of sample metadata, such as those matching host age or sex [43]. Such basic metadata associated with microbiome studies is also characterized by high levels of sparsity or missingness (e.g. >81.6% missing values for host age [43]). Although a number of resources and tools aimed at simplifying the annotation and formatting of sequence data and metadata exist (e.g. METAGENOTE [44]), their adoption is slow and these trends likely reflect the lack of centralized validation of standards or metadata quality during the submission process. As a result, even microbiome studies that share data in the INSDC databases (Bronze and above for DAS) often report only the minimally required metadata, which frequently falls short of minimum requirements for study-level reproducibility, lacks standardization, and contains free-text entries prone to inconsistencies and errors.

The misalignment between DAS and metadata reporting in terms of badge assignments in our case studies is concerning, as insufficient descriptive metadata directly limits the utility and future reuse of sequence data as they are interconnected [45]. Recognizing this issue, some initiatives are working on enriching microbiome data using text mining to extract relevant metadata terms from associated publications [46]. Such metadata rescue efforts can help when basic structured metadata are missing (e.g. geographic annotation), but retrospective mapping of more complex metadata terms remains challenging. To this purpose, our proposed metadata badge system may serve as a qualitative guide for metadata reporting and harmonization in the microbiome field. As our current publication offers a prototype of badge tiers for human gut-associated metadata, we hope future iterations will adapt these requirements for different fields.

### Ensure quality by (self-)evaluating data sharing practices

We note that the high proportion (>45%) of None-badged publications with statements of “data available upon reasonable request” is concerning, as such data accessibility statements can include ostensible promises of data, which often never materialize [33]. Overall, these findings are consistent with proportions of data availability previously reported by Tedersoo *et al.* (2021) [14], in which an average of only 54.2% articles had accessible data prior to contact.

Temporal patterns in data availability reveal encouraging trends (Fig. 3B). In the human fungal gut microbiome (mycobiome) studies, researchers have been adopting better data reporting practices at higher rates than in the mid-2010s (Supplementary Fig. S4), likely due to stricter peer-review monitoring of data submission over the last decade and the growing number of initiatives that encourage data sharing by adding visibility to accessible data [1]. Differences in data reporting guidelines [21] and the adoption rate of ORD standards (including variation in the peer-review process across journals) are also likely responsible for the apparent influence of a journal’s publisher on the distribution of assigned badges and data openness (Supplementary Fig. S5).

As a single binary standard is insufficient to cover a wide range of cases, we propose a sliding scale of specific and measurable tiers that reflects usability and ease to other researchers [4]. Our tiered “badge” system is designed to evaluate data reporting based on the level of information provided by authors (Graphical abstract): Bronze level contains the minimum required details for data accessibility and reuse, while Gold-ranked contributions allow for data access with little to no effort by users. Such a system provides coherent metrics to (self-)evaluate the quality of a dataset prior to investing time in reuse. In tandem with a user-friendly software for automated assessment, these unified standards and tier system will enable researchers, journals, and funding agencies to automatically assess compliance and quality of submitted datasets or publications, providing users with immediate, informative feedback [22, 47].

### Future directions

Our badge tier systems for sequencing data and metadata, and our associated prediction algorithm MISHMASH, are intended as proof of concepts. Their current iterations come with limitations, including a focus on the human gut microbiome in the given metadata badge standards. Future applications of metadata badge standards to nonhuman gut data should be adapted accordingly, possibly in the vein of MIxS standards applied to particular research fields. We note that due to heterogeneity in metadata reporting standards as described previously, the software MISHMASH does not currently provide metadata badge assessments. Future directions for improvement could include use of large language models to interpret and harmonize metadata.

While our prediction algorithm performs exceptionally well in establishing sequencing data availability (>97%; Table 3), incomplete semantic processing necessitates limited manual checks and validation. Most assignment errors can be attributed to the difficulty in discriminating between badge tiers as opposed to between None versus Badged publications, and can use continued improvement. In this initial proof-of-concept work, we were limited to databases with a robust API and thus were only able to evaluate open-access PMC publications available for web scraping (Fig. 1); similarly, sequence data were only programmatically assessed if found in INSDC databases and certain common databases such as the MG-RAST [48] or the Genome Sequence Archive of the Chinese National Genomics Data Center [42]. Although the PMC and INSDC do not encompass the entirety of scientific research, we expect our findings to be representative, as PMC and INSDC are among the largest and the most extensive research databases (Table 4). However, we hope that future iterations of our code will be able to automatically evaluate sequence data from less commonly used databases as well. We encourage the storage of sequence data in such databases rather than personal web pages due to issues with maintenance and “link rot” in the latter [47].

Our tier-based badge system promotes completely open data sharing, but an ethical issue arises in evaluating the accessibility of data locked under confidentiality agreements. Particularly with personally identifiable clinical data, patient confidentiality prevents completely public availability. Initiatives such as the European Genome-Phenome Archive (EGA) [49, 50] provide a workaround as a secure archive released to authorized researchers. While data within the EGA is currently outside the scope of this proof-of-concept work, we would support a future integration to extend badging assessments to publications associated with the EGA and similar databases.

Additional training and coordinated educational campaigns will be needed to overcome perceived barriers to sharing data, which should start by building trust and increasing awareness regarding the value of ORD for individual scientists, wider scientific community, and society at large [47]. Moreover, further user engagement will be needed to develop and extend this proof-of-concept resource to serve the specific needs of user communities across the microbiome research field [51]. While here we focus on microbiome research, our tier-based standards and software approach are extensible and these principles can be easily adopted by life sciences fields relying on similar data types, such as environmental DNA analysis [52] and diet-based DNA metabarcoding [53].

We hope that the release of our badge system and software will encourage self-reflection and greater engagement in FAIR practices in the microbiome community. Our vision for future implementation includes community outreach and engagement with stakeholders to encourage adoption of these or similar standards. We foresee the potential for integration with existing infrastructure to facilitate FAIR research awareness. Academic search engines such as the Swiss Library Service Platform, Web of Science, and Google Scholar include supplementary details with their search results; platforms such as these could include automated badge displays showcasing data availability with each hit to highlight commitment to FAIR principles. Additionally, these tiered data availability standards and software can be used by funding bodies or publishers to highlight and encourage data sharing practices among grantees or authors; e.g. publishers who adopt these standards could integrate MISHMASH as an automated workflow to assign a badge during the publication process, and display badges alongside articles on the journal website. The ISME Journal already incorporates open data badges [54, 55] to acknowledge and incentivize open science practices, demonstrating how a badge system could be implemented. Data sharing is required by a number of grants, institutions, and journals, but compliance can be challenging to manually check; this can be remedied with the use of automated software and quantifiable tiered standards.

## Conclusion

In parallel with other fields in the life sciences, microbiome research is an increasingly data-driven domain that relies on large-scale, high-dimensional datasets. Unrestricted access to sequence data and metadata is essential for scientific transparency and innovative reuse, and is therefore increasingly encouraged by the microbiome research community and required by many publishers and funding agencies. Despite the active development of data sharing standards and best practices in microbiome research, here we show that many studies still suffer from poor data accessibility and low-quality metadata, limiting efficient data reuse and constraining the potential benefits of meta-analyses and synthesis of published results. At least partly, this issue is due to the lack of mechanisms for assessing and validating data reporting practices and compliance with ORD reporting requirements, and lack of awareness of best practices among authors.

Preparing published sequence data and metadata for reuse is time-consuming and often inefficient due to the lack of validation methods. Here, we propose tier-based standards and contribute an open-source software package intended for assessment of sequence data and metadata sharing practices in microbiome research by a diverse range of downstream users. We hope that these standards and software will help to promote better practices in microbiome research by serving multiple purposes: (i) enabling authors to self-assess compliance with FAIR principles, thereby increasing awareness and adoption of these standards in the field, and (ii) providing data repositories, publishers, funding agencies, and other stakeholders with a prototype model to automatically assess compliance of submitted datasets or publications. Moreover, this functionality will also facilitate meta-analysis as automated predictions can (iii) help users with rapid and accurate identification of studies with minimally available sequence data, and thus datasets potentially suitable for reuse.

Poor standardization of shared data remains a critical issue across the life sciences [1]. We anticipate that evaluating data openness across publications will both promote data-sharing practices in the field and encourage greater community support and efforts toward FAIR ORD sharing and reporting. The sequence data and metadata badge systems we propose here are preliminary guides for reporting standards, to support efforts for improving FAIR literacy in the microbiome research domain and beyond.

## Supplementary Material

gkaf777_Supplemental_Files

## Data Availability

No new sequence data were generated for this study. PMC and INSDC accession IDs used are listed in the supplementary tables. Code used for analysis and figure generation can be found at https://zenodo.org/records/16039307.
